# Psychophysiological fidelity: A comparative study of stress responses to real and simulated clinical emergencies

**DOI:** 10.1111/medu.15155

**Published:** 2023-07-01

**Authors:** Russell Peek, Lee Moore, Rachel Arnold

**Affiliations:** ^1^ Department for Health University of Bath Bath UK; ^2^ Department of Paediatrics and Child Health Gloucestershire Hospitals NHS Foundation Trust Gloucester UK

## Abstract

**Introduction:**

Experiencing psychological stress may affect clinician performance in acute emergencies. While simulation is used extensively in healthcare education, it is unknown whether simulation effectively replicates the psychophysiological stress of real‐world conditions. Thus, this study explored whether measurable differences exist in psychophysiological responses to acute stress in simulated compared with real‐world clinical practice.

**Methods:**

In this within‐subjects observational study, stress appraisals, state anxiety and heart rate variability (HRV) were recorded during simulated and real‐world emergencies in a 6‐month training placement in neonatal medicine. Eleven postgraduate trainees and one advanced neonatal nurse practitioner participated. Mean (*SD*) participant age was 33 (8) years; and eight participants (67%) were female. Data were collected at rest and immediately before, during and 20 min after simulated and real‐world neonatal emergencies. In situ simulation scenarios were modelled on those used in accredited neonatal basic life support training. Stress appraisals and state anxiety were assessed using Demand Resource Evaluation Scores and the short State‐Trait Anxiety Inventory, respectively. High‐frequency power, a component of HRV associated with parasympathetic tone, was derived from electrocardiogram recordings.

**Results:**

Simulation was associated with greater likelihood of threat appraisal and higher state anxiety. High‐frequency HRV reduced from baseline in simulated and real‐world emergencies but recovered further towards baseline 20 min after simulated events. Possible explanations for the observed differences between conditions include participants' previous experiences and expectations of simulation and the effect of post‐simulation debrief and feedback.

**Discussion:**

This study identifies important differences in psychophysiological stress responses to simulated and real‐world emergencies. Threat appraisals, state anxiety and parasympathetic withdrawal are educationally and clinically significant, given their known associations with performance, social functioning and health regulation. While simulation may facilitate interventions aimed at optimising clinicians' stress responses, it is vital to confirm that outcomes transfer to real‐world clinical practice.

## Abstract 2

Do simulated emergencies recreate the psychological experience of real‐world clinical practice? They are certainly stressful, but perhaps in a different way.

## 1 INTRODUCTION

Simulation is used extensively in health professions education to facilitate learning and assessment in a safe, controlled environment.^1^ Despite considerable research into knowledge and skill acquisition through simulation,^2^ there are relatively few studies exploring learners' stress responses. Psychophysiological changes have been reported in learners during clinical simulation, but without comparison to matched, real‐world events.^3^, ^4^, ^5^ The importance of understanding stress responses in different conditions is illustrated by a study of anaesthetic procedural skills training, which found that a simulated environment failed to generate the same stress responses as those observed during simulation in a clinical environment.^6^ The accurate representation of real‐world stimuli is regarded as important in simulation design and effectiveness,^7^ but learner responses are rarely considered when conceptualising fidelity. Furthermore, educators vary in their beliefs about when, and under what conditions, psychological stress might benefit or harm learning.^8^, ^9^ If we do not understand the experience of stress in different learning environments, we risk failing to optimise the potential value of simulation in preparing for clinical practice.

The experience of psychological stress is the result of a transaction between an individual and their environment, with evidence that stress responses (e.g. increase in heart rate) are modulated by conscious and subconscious cognitive processing.^10^, ^11^ The biopsychosocial model of challenge and threat proposes that physiological stress responses are driven by largely subconscious appraisal of a potential stressor and personal resources available to cope with its demands.^12^, ^13^ A challenge state occurs if resources are appraised as meeting or exceeding situational demands, while a threat state results when perceived demands exceed resources.^12^ Challenge and threat states have been studied using self‐reports of perceived demands and resources and by observing specific patterns of cardiovascular reactivity in motivated performance situations.^13^, ^14^ A challenge state is characterised by relatively higher cardiac output and lower peripheral vascular resistance than a threat state^15^ and has been associated with superior performance in high‐pressure environments (e.g. elite sport, the military and healthcare).^16^, ^17^ Psychophysiological stress responses may be amenable to training, offering the potential to enhance performance in acutely stressful situations.^18^

In contrast to self‐report measures, cardiovascular monitoring can be carried out continuously, with minimal risk of bias (e.g. social desirability) and without redirecting attention from other tasks. Heart rate variability (HRV) is a natural phenomenon, reflecting variation in initiation and conduction of electrical depolarisation of the heart.^19^ Heart rhythm and contractility are adjusted under autonomic nervous system control, and HRV analysis can provide information about the relative activity of parasympathetic and sympathetic branches.^20^ The high‐frequency (HF) spectral band is associated with parasympathetic activity, whereas low‐frequency power has been attributed to sympathetic activity or the balance of autonomic activity.^21^, ^22^ It has been suggested that HRV responses to acutely stressful situations might reflect challenge and threat states, with a threat state linked with decreases in HF HRV.^23^, ^24^ Theoretical models of neurovisceral integration propose that higher parasympathetic tone is associated with better emotional and health regulation, social functioning and executive cognitive performance.^25^, ^26^, ^27^ HF power, as a correlate of parasympathetic tone, may also be predictive of attentional capacity and performance in tasks requiring cognitive control and short‐term memory.^28^, ^29^

As healthcare simulation aims to facilitate learning and performance enhancement that transfers to real‐world practice,^1^ it is important that simulation generates realistic and representative psychophysiological stress responses. This study therefore examined whether measurable differences exist in psychophysiological stress responses (i.e. challenge and threat states, state anxiety and HF HRV) to acutely stressful events in simulated compared with real‐world clinical conditions. A secondary aim was to empirically assess the postulated relationship between challenge and threat states and HF HRV.

## 2 STUDY DESIGN AND METHODS

Following institutional approval (EP19/20/064), advanced neonatal nurse practitioners and foundation, general practice and paediatric specialty trainees working in neonatal medicine in a large UK District General Hospital (~6000 births/year) were invited to participate in the study. Individuals taking regular prescription medications which affect HRV, or with cardiac pacemaker or vagal nerve stimulator devices, were excluded from participation. A power calculation based on the results of a study of acute assessment‐related stress suggested a minimum eight participants would be required to detect differences in HF HRV.^30^ Informed consent was obtained after participants received verbal and written information about the study. Specifically, participants were advised that the decision whether or not to take part would have no impact on their training, supervision or assessment. Data were collected during normal scheduled working shifts. Participants were asked to record perceptions of stress before and after attending births as a neonatal first responder (i.e. bleep holder) and before and after similar in situ training simulations. They were also asked to wear an electrocardiogram (ECG) monitor to record HRV during the shift. Participants were familiar with the environment and equipment through induction training and regular involvement in routine in situ simulation.

Simulation scenarios required participants to demonstrate basic newborn life support skills to manage thermal control, airway, breathing and circulation using an Advanced Life Support baby manikin (Laerdal Medical, Norway), as practised in local training and accredited courses.^31^ To replicate real‐world practice, participants received a handover briefing before simulation, providing clinical information such as gestation and labour history. Participants were told that performance in simulation would not be assessed, but debrief and verbal feedback would be offered afterwards. Simulation was facilitated by an experienced instructor, who is also a member of the senior neonatal medical team. The facilitator had no responsibility for summative assessment or progression decisions for study participants but was involved in routine clinical support, supervision and training on the neonatal unit.

Twelve participants took part in 61 events (10 simulations and 51 real‐world emergency calls). Mean (*SD*) participant age was 33 (8) years; eight (67%) participants were female. Mean (*SD*) experience since professional qualification was 6 (2) years. Participants represented neonatal team diversity, with four general practice trainees, three junior and four senior paediatric trainees and one advanced neonatal nurse practitioner. All participants had training in newborn life support and previous simulation experience. Clinical support was always available from a senior colleague. Participants recorded the intervention required after attending real‐world emergencies and reported the perceived realism of simulation scenarios (see Table S2).

**FIGURE 1:**
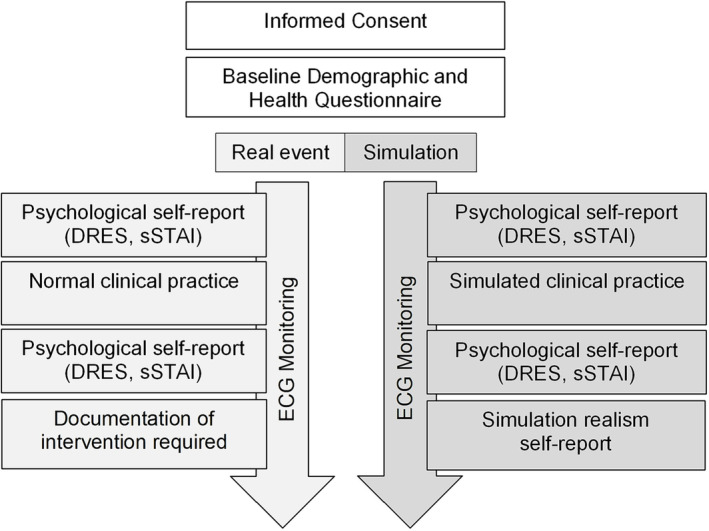
Visual illustration of the study protocol. DRES, Demand Resource Evaluation Score; ECG, electrocardiogram; sSTAI, short State‐Trait Anxiety Inventory. [Color figure can be viewed at wileyonlinelibrary.com]

Self‐report items were completed immediately before and after attendance for neonatal resuscitation or stabilisation or participation in a similar training simulation (see Figure 1). Items were completed in less than 2 min and did not interfere with routine clinical care. Specifically, participants reported challenge and threat appraisals using items adapted from the cognitive appraisal ratio.^14^, ^32^ Demand evaluations were assessed by asking “How demanding do you (or did you) expect the resuscitation or stabilisation to be?” rated on a 6‐point Likert scale from 1 (*not at all*) to 6 (*extremely*). Resource evaluations were measured on the same scale in response to the prompt “How able are you (or were you) to manage the clinical situation?.” Consistent with previous research,^33^ a Demand Resource Evaluation Score (DRES) was calculated by subtracting evaluated demands from resources (range −5 to +5), such that a negative value indicated a threat appraisal (i.e. situational demands exceed coping resources) and zero or a positive value indicated a challenge appraisal (i.e. resources match or exceed demands). The state scale of the short State‐Trait Anxiety Inventory (sSTAI) was used to assess state anxiety.^34^ The sSTAI includes six items (i.e. I feel … calm, tense, upset, relaxed, content and worried) measured on a 4‐point Likert scale from 1 (*not at all*) to 4 (*very much*). Calm, relaxed and content items were reversed, before item scores were summed and multiplied by a correction factor (20/6) for comparability with the full STAI state scale (range 20 to 80), where a larger value indicates higher state anxiety. The sSTAI is a valid and reliable indicator of state anxiety in a range of settings, including medical simulation.^3^, ^34^

Participants wore a three‐lead ECG monitor for continuous recording of heart rate and rhythm (Faros 180, Bittium Corporation, Finland). They were asked to press an event marker button on the ECG monitor when starting a simulation or attending a delivery. The monitor is lightweight and can be worn under scrubs or other clothing without interfering with normal activities. Five‐minute segments were analysed using Kubios software^35^ at baseline (i.e. rest), anticipation (i.e. after an emergency call but before treatment commenced), during the event, and recovery (i.e. 20 min after each event). Automatic artefact correction was checked with visual inspection of the ECG trace to confirm correct artefact identification.^36^ HF power was of particular interest, given its association with parasympathetic activity and evidence for parasympathetic withdrawal in acutely stressful situations.^21^, ^37^ Absolute HF power in the range 0.15–0.4 Hz was calculated using autoregressive modelling with a model order of 16.^36^ The natural logarithm transformation of absolute HF power (lnHF) was used in statistical analyses, as an estimate of vagal tone at normal breathing rates.^19^, ^38^

Data were analysed in R.^39^ After data cleaning, missing values were analysed for frequency and distribution. There was no missing DRES or sSTAI data. ECG recording artefact affected 18% of events. As this occurred unpredictably, data were treated as missing at random. Variables were analysed for central tendency and distribution, with potential outlying values identified using a *Z*‐score cut‐off of >3.29.^40^ Two events were associated with multiple outlying HRV values and were excluded from further analyses. Data were available from other events for these participants. Linearity of relationships between variables was assessed with scatterplots.

To explore potential differences between conditions (i.e. real‐world vs. simulated events), mean DRES and sSTAI were compared with independent *t*‐tests. Effect sizes were estimated with Hedges' *g*, with values of 0.2, 0.5 and 0.8 reflecting small, medium and large effects, respectively.^41^ Fisher's exact test was used to compare categorical challenge (DRES ≥ 0) and threat (DRES < 0) appraisals between conditions. Relationships between HF HRV, timepoint and condition were explored using a linear mixed effect model, with participant as the random effect. A mixed effects model allows for interpersonal variation in HRV, is appropriate to analysis of repeated measures data with missing values and is relatively robust to violations of distributional assumptions.^42^ Model fit was estimated using restricted maximum likelihood in the lme4 package for R.^43^ Analysis of deviance within models was performed using type III Wald F tests with Kenward–Roger degrees of freedom.^44^ QQ plots were examined to confirm normality of residuals. Results are reported as β estimates representing mean HF HRV with 95% confidence intervals. Planned post hoc pairwise comparisons were made with Holm–Bonferroni corrections. Pearson product–moment correlation coefficients were used to explore potential relationships between DRES and sSTAI in anticipation and recovery. Finally, differences in sSTAI and HF HRV between challenge (DRES ≥ 0) and threat (DRES < 0) groups were compared with independent *t*‐tests.

## 3 RESULTS

**FIGURE 2:**
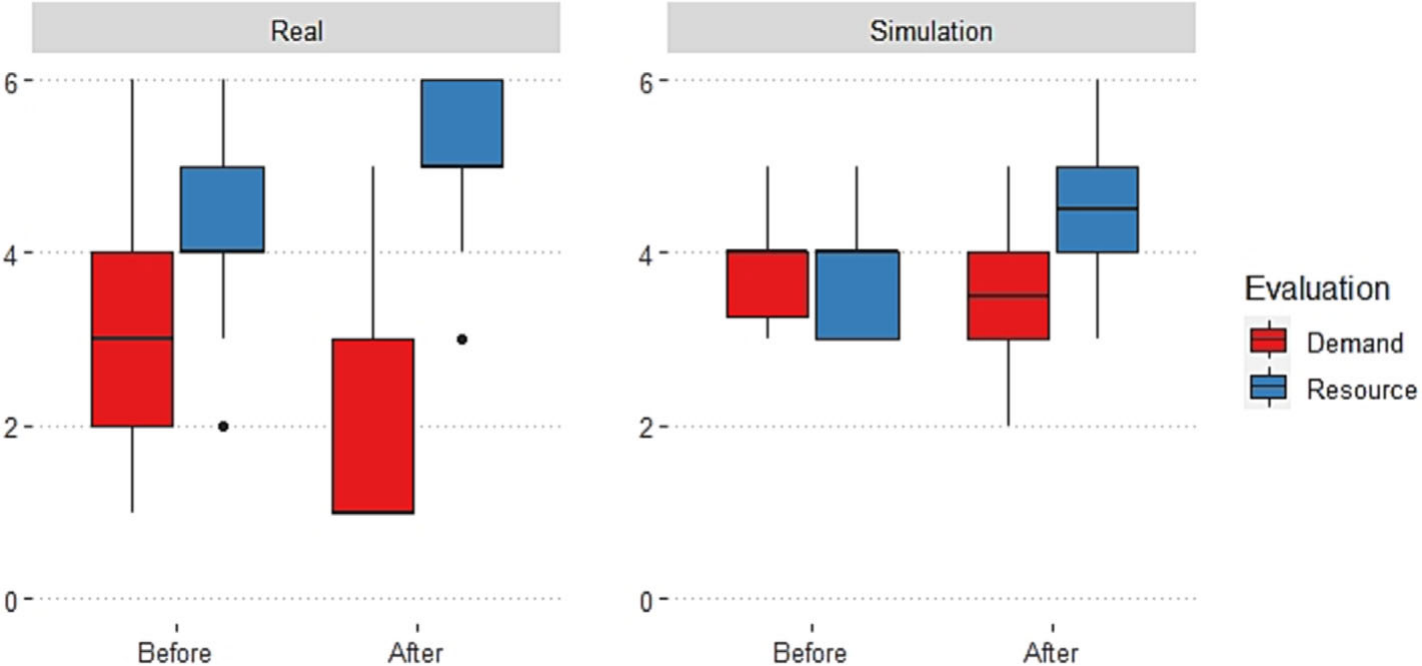
Boxplot of demand and resource evaluations by condition and timepoint. [Color figure can be viewed at wileyonlinelibrary.com]

In both real‐world and simulated conditions, DRES was higher after events than beforehand. In simulation, this change was driven primarily by a re‐evaluation of personal coping resources rather than a reduction in evaluated situational demands (Figure 2). In addition, DRES was significantly lower in simulation than in real‐world conditions (Table 1). Threat appraisals were more likely after simulated events than in real‐world conditions (Fisher's exact test, p = 0.028). State anxiety was higher in simulation compared with real‐world conditions at both timepoints, although this difference was not statistically significant (Table 2).

**TABLE 1:** DRES before and after events.

Timepoint	DRES mean (*SD*)					
	Real *n* = 51	Simulation *n* = 10	Mean difference	95% CI	p	Effect size (*g*)
Before	0.88 (1.76)	−0.10 (0.88)	0.98	0.22 to 1.74	0.014	0.58
After	3.33 (1.86)	1.00 (1.76)	2.33	1.00 to 3.66	0.002	1.25

Abbreviations: CI, confidence interval; DRES, Demand Resource Evaluation Score.

**TABLE 2:** sSTAI before and after events.

Timepoint	sSTAI mean (*SD*)					
	Real *n* = 51	Simulation *n* = 10	Mean difference	95% CI	p	Effect size (*g*)
Before	36.99 (12.21)	42.33 (8.17)	5.34	−1.17 to 17.98	0.102	0.45
After	33.53 (8.86)	36.67 (7.02)	3.14	−2.28 to 8.55	0.236	0.36

Abbreviations: CI, confidence interval; sSTAI, short State‐Trait Anxiety Inventory.

**FIGURE 3:**
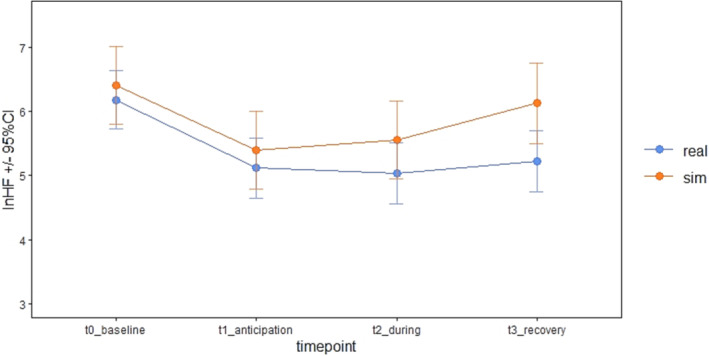
Mixed model interaction plot: High‐frequency heart rate variability, timepoint and condition. CI, confidence interval. [Color figure can be viewed at wileyonlinelibrary.com]

In real‐world and simulated conditions, HF HRV reduced from baseline to anticipation (Figure 3). It remained relatively stable during events, before increasing in recovery, particularly in simulated conditions. The mixed effects model of HF HRV demonstrated a statistically significant effect of timepoint (*F*_3,175_ = 22.02, p < 0.001), but not condition (*F*_1,176_ = 0.74, p = 0.390) (Table 3). However, planned post hoc simultaneous tests for general linear hypotheses demonstrated a statistically significant difference in HF HRV between real‐world and simulated conditions in recovery (*p* = 0.031), such that HF HRV was higher and closer to baseline in recovery after simulation.

**TABLE 3:** Mixed effects model, high‐frequency heart rate variability (mean lnHF) by timepoint and situation.

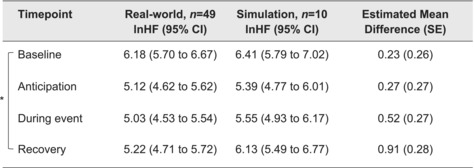

Abbreviation: CI, confidence interval.

*p < 0.001.

There was a significant moderate negative correlation between DRES and sSTAI, *r*(120) = −0.61, p < 0.001. State anxiety was significantly lower in participants appraising events as a challenge (mean [*SD*] STAI 33.5 [8.88]) rather than a threat (50.0 [7.23]), mean difference 16.5 (95% CI 12.5 to 20.4, p < 0.001). In relation to the secondary aim of this study, there was no statistically significant correlation between DRES and HF HRV and no significant difference in HF HRV between participants appraising events as a challenge or threat. Finally, it is worth noting that simulation scenarios were evaluated as realistic by participants (see Table S2). Moreover, the clinical intervention required in real‐world events and simulated scenarios was similar, with no infant requiring advanced life support (i.e. no intervention beyond thermal control and airway management, breathing support and cardiac compressions).

## 4 DISCUSSION

This study demonstrated significant differences in psychophysiological stress responses to simulated versus real‐world acute clinical events. Simulated events were associated with increased likelihood of threat appraisal (i.e. situational demands exceed personal coping resources) and greater state anxiety. Interestingly, mean state anxiety in anticipation of simulation was above the threshold often considered to indicate clinically significant anxiety (i.e. >39).^45^, ^46^ The observed association between threat appraisal and state anxiety is in keeping with theory and prior research.^33^, ^47^

A criticism often levelled at laboratory‐based psychophysiological studies is the possibility of a strong observer effect.^48^ Participants in the current study were effectively unobserved in real‐world practice, other than continuous ECG recording which provided no direct feedback. In contrast, simulation involved observation and interaction with a facilitator. In an ethnographic study, student paramedics regarded all simulation as assessed in some way, even if explicitly not designed or used for assessment, as in the current study.^49^ Thus, an observer effect or implicit association with assessment may underlie the stress appraisal and state anxiety responses to simulated events. Indeed, threat appraisals are more likely to occur when an individual feels socially stigmatised, is interacting with individuals with perceived higher status or when the interaction violates expectations.^50^, ^51^ Studies have shown that uncontrollable or socio‐evaluative stressors where others could negatively judge performance are most likely to provoke a threat response.^52^ This challenges the assumption that simulation provides a “safe space” in which to gain confidence and develop personal resources to apply in real‐world practice. It would be interesting to explore whether peer‐facilitated simulation^53^ affects stress appraisals by altering the observer–participant dynamic.

Experiencing challenge or threat states can influence component processes of motor performance, such as visual attentional control and muscular activity, and thus affect overall performance quality.^54^ In a threat state, individuals may invest greater effort in self‐regulation, drawing resources from a finite pool for managing thought, emotion and behaviour.^55^ Considering the visuomotor skills required to successfully manage the resuscitation of critically ill patients, it is relevant to note that individuals who consciously focus on regulating motor skill execution (a process known as reinvestment) perform poorly.^56^ Furthermore, emotions such as anxiety can contribute to learner cognitive load during simulation.^5^ The findings of this study therefore have implications for the use of simulation in high‐stakes competence‐based assessment. If simulation causes threat appraisals and greater anxiety, performance in simulation may not provide an accurate reflection of competence in real‐world emergencies.

The drop in HF HRV from baseline in real‐world and simulated conditions is also relevant to performance, given the positive association between parasympathetic tone and social functioning, attentional control, cognition and short‐term memory.^25^, ^28^, ^29^ Each of these functions is important to providing effective emergency medical care.^57^ The change in HF HRV from baseline to anticipation and from anticipation to during events was similar across conditions, but HF HRV returned further towards baseline after simulated events than real‐world emergencies. HF HRV was only assessed and analysed at a single timepoint after events, but this raises the interesting question of whether physiological consequences of acutely stressful situations persist for longer in real‐world settings and, if so, for how much longer. The possibility of a persisting effect is particularly relevant to intense environments with frequent acutely stressful events and little time for recovery in‐between. Intervention strategies to optimise psychophysiological stress responses (e.g. arousal reappraisal) might be particularly beneficial in such workplaces.^58^

Observed patterns of change in HF HRV may reflect differential activation of sympathetic–adrenomedullary (SAM) and hypothalamic–pituitary–adrenocortical (HPA) axes.^59^ The SAM response is fast in onset, consistent with the initial change observed in both conditions. SAM activation may be adaptive to the demands of a medical emergency. There is evidence that acute psychophysiological stress responses can facilitate stimulus–response learning.^60^ Because resuscitation training programmes aim to teach a shared, algorithmic approach to life support, a SAM response may be beneficial to the learning and retention of these skills. HPA responses are slower in onset, with cortisol peaking 20–40 min after the onset of a stressful event.^59^ Recovery to baseline is also prolonged, taking 40–60 min after resolution of the stressor. The sustained HF HRV changes in real‐world conditions might imply prolonged SAM activation or greater HPA axis activity. This is potentially maladaptive, given the long‐term impact of cumulative stress on immune function and health.^61^

An important difference between real‐world and simulated conditions was the use of feedback and debrief after simulation. While the primary intention was educational,^62^ it is possible that approaches to facilitate reflection and experiential learning also support recovery and restoration of HF HRV. Simulation debrief typically involves several phases, including opportunities to discuss emotional responses, thought processes and decision making, as well as the knowledge and skills underpinning successful management of the situation.^63^ Interestingly, a similar pattern of change in response to simulation and debrief has been reported previously, with recovery of tranquillity (i.e. a positive, low arousal state) observed in medical students after debriefing.^5^ Unless there is a poor or unexpected outcome, debrief is not routinely carried out after attending emergency calls in real‐world practice. Investigating the effect of debrief on psychophysiological responses to acutely stressful real‐world events would add to the findings of the current study. Real‐world emergencies occurred with greater frequency than simulation during this study. It is thus possible that greater familiarity with real‐world conditions might have contributed to the observed differences (e.g. lower anxiety in real‐world events). Furthermore, simulation was planned and could therefore be anticipated, while real‐world events occurred unpredictably with little notice. However, previous research found no difference in perceived stress between unannounced and routinely planned simulation.^64^

A major strength of this study is the within‐subjects design, which allowed comparison of psychophysiological stress responses in real‐world and simulated conditions while controlling for potential inter‐individual differences in HRV due to factors including age, physical activity and fitness.^36^ Challenge and threat appraisals and state anxiety were recorded prospectively, with the same instruments used in real‐world and simulated conditions. Simulation scenarios were designed and evaluated to ensure they reflected real‐world clinical care as closely as possible. Potential limitations include that the study was designed to identify whether measurable differences exist in psychophysiological responses to real‐world and simulated events, rather than to explore causation. Because psychophysiological stress responses involve interacting systems (e.g. SAM and HPA), observing systems in isolation will give a relatively limited understanding of the impact of psychophysiological phenomena on health.^61^ In this study, challenge and threat appraisals and HF HRV provided complimentary but differing views of psychophysiological stress responses. The addition of other biomarkers, such as salivary cortisol, would provide a more comprehensive picture of whether stress responses differ between simulated and real‐world emergencies.^65^ Future research could also usefully explore whether challenge and threat appraisals in anticipation of clinical emergencies are amenable to intervention (e.g. arousal reappraisal).^58^, ^66^

## 5 CONCLUSION

This study compared psychophysiological responses to acutely stressful events in real‐world and simulated conditions in early‐career postgraduate clinicians. The observed differences in challenge and threat appraisals, state anxiety and HF HRV are relevant to healthcare education, clinical performance and learner health and well‐being. Participant responses should be considered an important component of simulation fidelity. Educators should consider whether it is possible to design simulation training so that the psychological experience is more closely matched to real‐world clinical practice. Further research might usefully explore whether peer‐facilitated simulation or other interventions (e.g. arousal reappraisal) result in more optimal psychophysiological stress responses (e.g. akin to a challenge state). While simulation may facilitate interventions aimed at optimising clinicians' stress responses, it is vital that researchers confirm that outcomes transfer to real‐world clinical practice.

## AUTHOR CONTRIBUTIONS

**Russell Peek:** Conceptualization; investigation; writing—review and editing; writing—original draft; formal analysis; methodology. **Lee Moore:** Writing—review and editing; methodology. **Rachel Arnold:** Writing—review and editing; methodology.

## CONFLICT OF INTEREST STATEMENT

The authors have no conflicts of interest to declare.

## ETHICS STATEMENT

The study was reviewed and approved by the University of Bath Research Ethics Approval Committee for Health (REACH), EP19/20/064. Local site approval was received from the Gloucestershire Hospitals NHS Foundation Trust Research and Development Department. All work was conducted in accordance with relevant ethical guidelines and principles.

## Supporting information


**Table S1.** HRV data by timepoint and condition, *N* = 59 events.
**Table S2.** Participant evaluation of simulation realism.

## Data Availability

The data that support the findings of this study are available from the corresponding author upon reasonable request.
