# Supplementary material for: Psychophysiological fidelity: A comparative study of stress responses to real and simulated clinical emergencies
Source: Med Educ. 2023 Jul 1;57(12):1248–56. doi: 10.1111/medu.15155 (PMC10946833; doi:10.1111/medu.15155)
Supplement: Supplementary file 1 — Table S1. HRV data by timepoint and condition, N = 59 events. Table S2. Participant evaluation of simulation realism. [file MEDU-57-1248-s001.docx]

**Supplementary material.**

**Table i.** HRV data by timepoint and condition, *N*=59 events.

|  |  | **Real, n=49** | | **Simulation, n=10** | |
| --- | --- | --- | --- | --- | --- |
| **Item** | **Timepoint** | Mean (*SD*) | Missing (%) | Mean (*SD*) | Missing (%) |
| **Heart period** | Baseline | 839.35 (133.66) | 0 (0.00) | 864.61 (128.67) | 0 (0.00) |
| (ms)* | Anticipation | 650.84 (104.37) | 13 (26.53) | 692.42 (83.60) | 0 (0.00) |
|  | During | 652.78 (104.58) | 14 (28.57) | 663.14 (95.23) | 0 (0.00) |
|  | Recovery | 714.50 (121.70) | 15 (30.61) | 759.34 (92.49) | 1 (10.00) |
| **RMSSD** | Baseline | 44.28 (25.01) | 0 (0.00) | 57.16 (29.30) | 0 (0.00) |
|  | Anticipation | 21.12 (9.76) | 13 (26.53) | 30.87 (12.73) | 0 (0.00) |
|  | During | 20.67 (9.16) | 14 (28.57) | 36.61 (27.32) | 0 (0.00) |
|  | Recovery | 23.24 (9.87) | 15 (30.61) | 37.24 (13.62) | 1 (10.00) |
| **pNN50** | Baseline | 20.01 (19.77) | 0 (0.00) | 29.90 (21.99) | 0 (0.00) |
|  | Anticipation | 3.79 (5.49) | 13 (26.53) | 9.81 (7.12) | 0 (0.00) |
|  | During | 3.45 (3.79) | 14 (28.57) | 10.35 (9.78) | 0 (0.00) |
|  | Recovery | 5.32 (6.24) | 15 (30.61) | 13.60 (9.62) | 1 (10.00) |
| **ln LF** | Baseline | 7.10 (0.70) | 0 (0.00) | 7.25 (0.74) | 0 (0.00) |
|  | Anticipation | 6.55 (0.75) | 13 (26.53) | 6.86 (0.60) | 0 (0.00) |
|  | During | 6.61 (0.91) | 14 (28.57) | 6.85 (0.81) | 0 (0.00) |
|  | Recovery | 6.56 (0.80) | 15 (30.61) | 7.08 (0.64) | 1 (10.00) |
| **ln HF** | Baseline | 6.13 (0.88) | 0 (0.00) | 6.72 (0.99) | 0 (0.00) |
|  | Anticipation | 4.90 (0.82) | 13 (26.53) | 5.71 (1.10) | 0 (0.00) |
|  | During | 4.83 (1.06) | 14 (28.57) | 5.87 (1.15) | 0 (0.00) |
|  | Recovery | 5.00 (0.91) | 15 (30.61) | 6.41 (0.85) | 1 (10.00) |
| **LF/HF ratio** | Baseline | 4.00 (3.84) | 0 (0.00) | 2.94 (4.10) | 0 (0.00) |
|  | Anticipation | 6.58 (4.22) | 13 (26.53) | 4.50 (4.58) | 0 (0.00) |
|  | During | 7.57 (5.03) | 14 (28.57) | 3.77 (3.56) | 0 (0.00) |
|  | Recovery | 6.54 (4.92) | 15 (30.61) | 4.17 (4.33) | 1 (10.00) |

Note: *Heart Rate can be calculated by dividing 60 000 by Heart period.

**Table ii.** Participant evaluation of simulation realism.

| **Item** | **Median score (IQR)** |
| --- | --- |
| The physical environment felt realistic | 3.00 (3.00-3.25) |
| I am familiar with the clinical equipment used | 4.00 (4.00-4.00) |
| The background situation or scenario felt genuine | 4.00 (4.00-4.00) |
| The scenario played out in a believable way | 3.50 (3.00-4.00) |
| I felt I was in my normal role for the simulation | 4.00 (4.00-4.00) |
| I could assess the clinical situation as I usually would | 4.00 (3.00-4.00) |
| Getting feedback about the effect of my actions felt realistic | 3.00 (3.00-4.00) |
| Interaction with other team members felt normal | 3.50 (3.00-4.00) |
| I was engaged with the simulation | 4.00 (4.00-4.00) |
| I had to use a lot of imagination | 1.00 (1.00-2.00) |

Note: Likert scale range = 0 (not at all) to 4 (very much)
